# Avenanthramide C suppresses hypoxia-induced cyclooxygenase-2 expression through sirtuin1 activation in non-small-cell lung cancer cells

**DOI:** 10.1080/19768354.2020.1748108

**Published:** 2020-04-02

**Authors:** Wonchung Lim, Chounghun Kang

**Affiliations:** a Department of Physical Education, Inha University, Incheon, South Korea; b Department of Sports Medicine, College of Health Science, Cheongju University, Cheongju, South Korea

**Keywords:** Avenanthramide C, hypoxia, cyclooxygenase-2, SIRT1

## Abstract

Avenanthramide C (AVC), found mainly in oats, mediates anti-inflammatory activities by reducing the anti-inflammatory cytokine levels. This study investigated the effects of AVC on hypoxia-induced cyclooxygenase-2 (COX-2) expression in A549 cells. AVC suppressed the hypoxia-induced increase in COX-2 protein levels and promoter activity. We also observed that the effects of AVC were reversed by a SIRT1 inhibitor, indicating that the inhibitory effects of AVC on hypoxia-induced COX-2 expression are mediated by SIRT1. Therefore, AVC inhibits the hypoxic induction of COX-2 expression via SIRT1 activation. Our results suggest that AVC could be beneficial for preventing lung inflammation under hypoxia.

## Introduction

Inflammatory reactions are common in the lungs and they result in the necrosis and fibrosis of the lung tissue that has poor pathological prognosis (El Rayes et al. 2015). Among the several causes of inflammatory reactions, hypoxia is known to be a significant cause of lung inflammation (Tuleta et al. 2016). Hypoxia may result in the induction of cyclooxygenase-2 (COX-2) in various conditions (Kaidi et al. 2006; Fredenburgh et al. 2009; Lee et al. 2010; Zhao et al. 2012). Furthrmore, COX-2 and prostaglandin E2 (PGE2) play a pivotal role in inflammatory responses and metastasis (Xiong et al. 2005; Moore et al. 2009).

Avenanthramides (AVs), found exclusively in oats, are made up of an anthranilic acid derivate and a hydroxycinnamic acid derivate linked by a pseudo-peptide, (Collins 1989) and have anti-inflammatory effects (Koenig et al. 2016). Koenig *et al* demonstrated that AV supplementation attenuates the eccentric exercise-inflicted blood inflammatory markers in women (Koenig et al. 2016). AV attenuates exercise-induced inflammation in postmenopausal women, (Koenig et al. 2014) which coincides with the finding that AV has antioxidant activity in humans (Fu et al. 2015). One of the mechanisms by which AV exerts its anti-inflammatory effects is through suppression of the nuclear factor kappa B (NF-κB) signaling pathway (Guo et al. 2008) by inhibiting IκB-α degradation and decreasing phosphorylation of p65 (Sur et al. 2008).

However, so far there is no evidence that AV affects hypoxia signaling. Furthermore, the mechanism by which AV inhibits the expression of COX-2 during hypoxia has not been elucidated. In this study, we found that AVC inhibits hypoxia induction of COX-2 that is mediated by sirtuin 1 (SIRT1), a deacetylating protein that contributes to cellular regulation. Our results provide new insights into the mechanisms by which AVC affects the inflammatory response in lung cancer cells under hypoxia.

## Materials and methods

### Materials

AVA, AVB, and AVC were purchased from Sigma (St. Louis, MO, USA). Fetal bovine serum (FBS), Trizol Reagent, and penicillin/streptomycin were purchased from GIBCO Invitrogen (Grand Island, NY, USA). Anti-β-actin was purchased from Sigma (St. Louis, MO, USA). Anti-COX-2 was purchased from Santa Cruz Biotechnology (Santa Cruz, CA, USA).

### Cell culture and hypoxic conditions

Human pulmonary epithelial A549 cells were maintained in DMEM containing 10% FBS and penicillin/streptomycin. Cells were grown at 37°C in a humidified atmosphere of 95% air/5% CO_2_ and fed every 2–3 days. Before treatment, the cells were washed with phosphate-buffered saline and cultured in DMEM/5% charcoal–dextran stripped FBS (CD-FBS) for 2 days. All treatments were done with DMEM/5% CD-FBS. For the hypoxic condition, cells were incubated with a mixture of 1% O_2_, 5% CO_2_, and 94% N_2_ using a hypoxic chamber (Thermo Fisher Scientific**,** Waltham, MA, USA).

### Plasmids

COX-2-Luc, a firefly luciferase reporter construct containing the human COX-2 gene promoter fragment −327/+59, was kindly provided by Dr. Hiroyasu Inoue (Nara Women’s University, Nara, Japan).

### Transfection and luciferase assays

A549 cells were transiently transfected with plasmids using polyethylenimine (PEI; Polysciences, Warrington, PA, USA). The luciferase activity was determined 24 or 48 h after treatment using the luciferase assay system (Promega Corp., Madison, WI, USA) with an AutoLumat LB9507 luminometer (EG & G Berthold, Bad Widbad, Germany) and expressed in relative light units.

### Western blot analysis

Proteins were isolated using lysis buffer (150 mM NaCl, 50 mM Tris-HCl, 5 mM EDTA, 1% Nonidet P-40, 0.5% deoxycholate, 1% SDS) with a protease inhibitor cocktail (Sigma, St. Louis, MO, USA) on ice for 1 h and then centrifuged for 20 min at 13,000 × g. The supernatant was collected, and the protein concentrations were measured using the Bradford method (Bio-Rad, Hercules, CA, USA). Proteins were dissolved in sample buffer and boiled for 5 min prior to loading onto an acrylamide gel. After SDS-PAGE, the proteins were transferred to a polyvinylidene difluoride membrane, blocked with 5% nonfat dry milk in Tris-buffered saline containing 0.1% Tween-20 (TBST) for 60 min at room temperature. The membranes were incubated for 2 h at room temperature with the primary antibody. Equal lane loading was assessed using actin monoclonal antibody (Sigma, St. Louis, MO, USA). After washing with TBST, the blots were incubated with 1:5000 dilution of the horseradish peroxidase conjugated-secondary antibody (Invitrogen, Grand Island, NY, USA), and washed again three times with TBST. The transferred proteins were visualized with an enhanced chemiluminescence detection kit (Amersham Pharmacia Biotech, Buckinghamshire, UK).

### Statistical analysis

Data were expressed as mean ± SD, and statistical analysis for single comparison was performed using the Student’s *t*-test. The criterion for statistical significance was *P* < 0.05.

## Results

**Figure 1.**
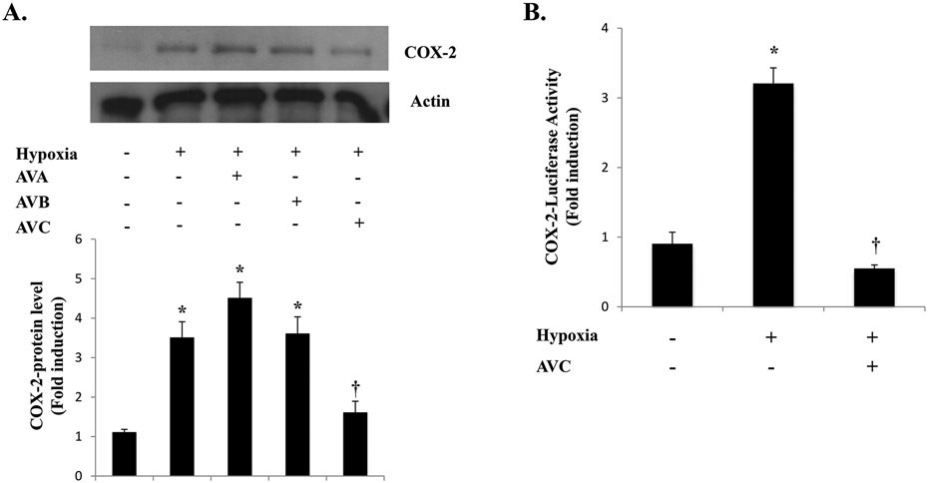
AVC inhibits hypoxia-induced COX-2 expression in A549 cells. (A) A549 cells were pretreated with the indicated concentrations of AVs for 1 h before treatment with hypoxia for 24 h and were analyzed by western blotting, (B) A549 cells were transfected with COX-2-Luc and treated as indicated. After treatment, luciferase expression was determined as described in the Materials and Methods. Values represent the mean ± SD (N = 3). *P < 0.05. All experiments were repeated at least three times.

**AVC inhibits hypoxia induction of COX-2 expression in A549 cells** COX-2 overexpression mediates both tumor cell survival and angiogenesis under hypoxia (Kaidi et al. 2006). However, there have been no reports on the effect of AVs on COX-2 expression under hypoxia in lung cancer cells. To assess the effects of AVs on hypoxia induction of COX-2 in A549 cells, we pre-incubated cells with avenanthramides for 1 h and subjected them to hypoxia. At 100 μM, AVC efficiently blocked the hypoxia-induced COX-2 protein expression (Figure 1(A)). AVC treatment at 100 μM also inhibited hypoxia-induced COX-2 promoter activity (Figure 1(B)). These results indicate that AVC inhibits hypoxia-induced COX-2 regulation in A549 cells.

**Figure 2.**
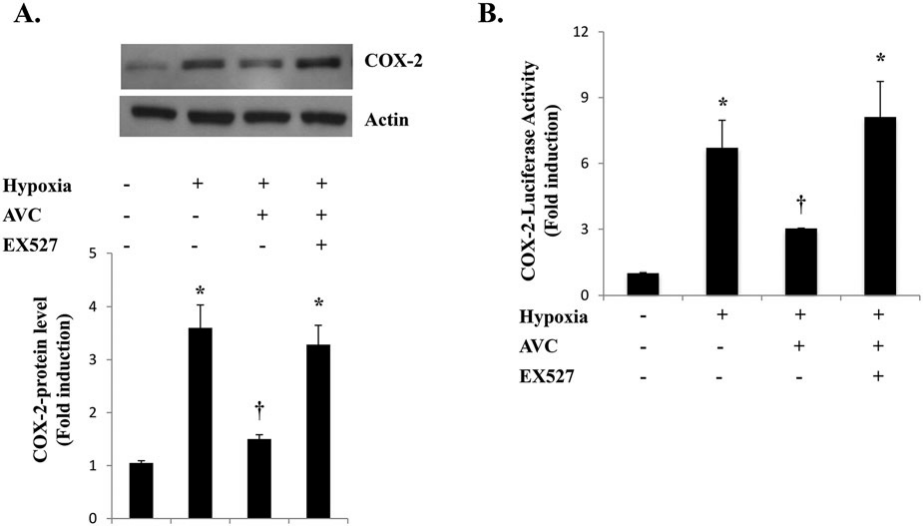
SIRT1 activation is required for the AVC-mediated inhibition of hypoxia-induced COX-2 expression. (A) A549 cells were pretreated with AVC (100 μM) and/or EX527 (1 μM) for 1 h before treatment with hypoxia for 24 h and were then analyzed by western blotting, (B) A549 cells were transfected with COX-2-Luc reporter and treated as indicated. After treatment, the luciferase activity was assayed. Values represent the mean ± SD (N = 3). *P < 0.05, **P < 0.01. All experiments were repeated at least three times.

**SIRT1 activation is required for the AVC-mediated inhibition of hypoxia-induced COX-2 expression** A recent study indicates that SIRT1 plays an important role in the suppression of inflammation (Lim et al. 2015). To determine whether SIRT1 activation is involved in the COX-2 inhibition by AVC, we measured the COX-2 protein levels following cotreatment with a SIRT1 specific inhibitor, EX527. The inhibition of hypoxia-induced COX-2 expression by AVC was abolished by EX527 cotreatment (Figure 2(A)). Similarly, no inhibition of COX-2 promoter activity by AVC was observed when EX527 was administered (Figure 2(B)). These results indicate that SIRT1 mediates the inhibitory effects of AVC in the hypoxia-induced increase in COX-2.

**Figure 3.**
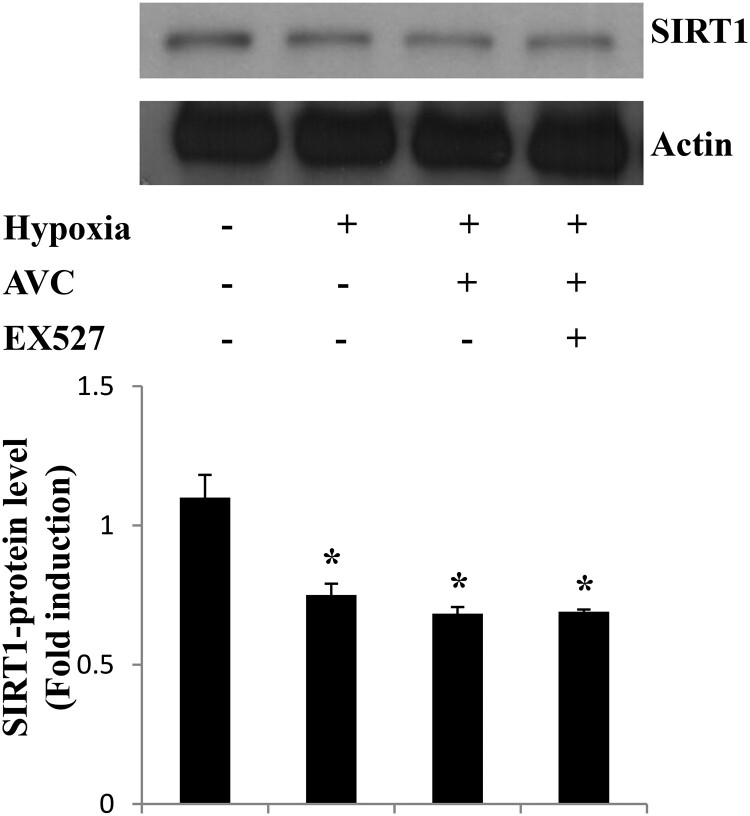
Alterations in SIRT1 protein levels are not necessary for the AVC-mediated inhibition of hypoxia-induced COX-2 expression. A549 cells were treated as indicated and analyzed by western blotting. Values represent the mean ± SD (N = 3). *P < 0.05. All experiments were repeated at least three times.

**Alteration of SIRT1 protein levels is not necessary for the AVC-mediated inhibition of hypoxia-induced COX-2 expression** Korean red ginseng exerts anti-inflammatory activity under hypoxia through SIRT1 activity and protein levels (Lim et al. 2015). Here, we investigated whether AVC could affect SIRT1 protein levels under hypoxia. As shown in Figure 3, hypoxia resulted in a downregulation in SIRT1 protein expression, which was not reversed by AVC, suggesting that alteration of SIRT1 protein levels is not necessary for the AVC-mediated inhibition of hypoxia-induced COX-2 expression in A549 cells.

## Discussion

The COX-2 plays a crucial role in the pathogenesis of lung inflammation (Rumzhum and Ammit 2016). Overexpression of COX-2 is associated with the development of adenocarcinomas and with lung metastasis (Hida et al. 1998). COX-2 inhibitors have been tested in clinical trials for lung cancer (Liu et al. 2015). In cancer pathology, it has been reported that SIRT1 regulates tumor growth (Shin et al. 2016). A recent study showed that SIRT1 regulates the hypoxia-induced resistance of non-small cell lung cancer to cisplatin and doxorubicin (Shin et al. 2014). Although few previous studies have reported the effect of AV on inflammation, there is no direct evidence for its effect under hypoxia. Therefore, further studies on the detailed molecular mechanism underlying the inhibitory effect of AVC on the increase in COX-2 in lung cancer cells under hypoxia are necessary. In this study, hypoxia markedly increased COX-2 expression. Pretreatment with AVC inhibited the hypoxia-induced increased COX-2 expression. It was observed that the suppression of hypoxia-induced COX-2 expression by AVC was attenuated by EX527 in A549 cells (Figure 2). Recent reports indicate that diverse hypoxia-induced biological effects are correlated with SIRT1. The depletion of energy in hypoxia-initiated cell death pathways leads to the downregulation of SIRT1 (Meng et al. 2017). SIRT1 activation was required for the AVC effects under hypoxia, although the levels of SIRT1 protein were not altered. These results indicate that SIRT1 enzymatic activity seems to be more important than SIRT1 protein levels for AVC action under hypoxia.

SIRT1 protects from K-Ras-driven lung carcinogenesis (Costa-Machado et al. 2018). The abnormal expression of SIRT1 was involved in the development of non-small-cell lung cancer (Yang 2018). SIRT1 plays a protective role against inflammatory lung injury (Gao et al. 2015). Improving the expression and activity of SIRT1 through calorie restriction and resveratrol treatment could play a protective role in hypoxic conditions (Meng et al. 2017). However, the potential role of SIRT1 on the AVC-mediated anti-inflammatory effect in lung tissue during tumor development remains elusive. In our system, AVC may affect NAD^+^/SIRT1/NF-κB axis in our system. AVC may increase the total amount of NAD^+^ in A549 cells, which could activate SIRT1 to downregulate acetylated NF-κB. Previous studies have shown that AVC is an allosteric protein–protein inhibitor for modulating IKKβ’s affinities (Kang et al. 2018) and inhibits the phosphorylation of IKK and IκB (Guo et al. 2008). Thus, AVC could reduce the level of acetylated NF-κB by upregulation of NAD^+^ levels and the expression of COX-2 in lung tissues. To our knowledge, this is the first study showing that AVC has a strong suppressive effect on COX-2 expression under hypoxia.

Although a recent study showed that AV downregulated the expression of the hypoxia-inducible factor-1α (HIF-1α) gene in cancer cell lines, (Kang et al. 2018) the effects of AVC on hypoxic signaling has not been investigated so far. HIF-1α has a critical role in hypoxia; (Guo et al. 2008) however, we did not examine the involvement of HIF-1α signaling in this study. Thus, further studies are needed to elucidate the detailed mechanism underlying AVC action.

Until now, little attention has been focused on the effects of AVC in hypoxia-induced inflammation. Our results suggest that AVC inhibits hypoxia-induced COX-2-expression via SIRT1 in A549 cells. This inhibition of COX-2 probably contributes to the anti-inflammatory and chemopreventive properties of AVC under hypoxic conditions.
